# Analysis on Changes and Influencing Factors of the Intestinal Microbiota of Alpine Musk Deer between the Place of Origin and Migration

**DOI:** 10.3390/ani13243791

**Published:** 2023-12-08

**Authors:** Baofeng Zhang, Minghui Shi, Shanghua Xu, Haonan Zhang, Yimeng Li, Defu Hu

**Affiliations:** 1 School of Ecology and Nature Conservation, Beijing Forestry University, Beijing 100083, China; 2 Guangxi Forestry Research Institute, Nanning 530002, China; 3 Department of Life Sciences, National Natural History Museum of China, Beijing 100050, China

**Keywords:** Alpine musk deer, intestinal microbiota, place of origin, place of migration

## Abstract

**Simple Summary:**

The intestinal microbiome structure and diversity of Alpine musk deer in Gansu (origin) and Sichuan (migration) were analyzed in this study using 16S rRNA high-throughput sequencing technology. The study revealed that there were no alterations in the dominant intestinal species between musk deer in the origin and migration areas. However, there were significant differences in their relative abundance. Therefore, specific measures should be implemented for the feeding and management of migratory Alpine musk deer. This study provides a theoretical foundation for expanding artificial populations and reintroducing wild populations.

**Abstract:**

In China, the population of wild musk deer, belonging to the family Moschidae, has drastically decreased in recent years owing to human activities and environmental changes. During the 1990s, artificial breeding of Alpine musk deer was conducted in Xinglong Mountain, Gansu Province, China, and their ex situ conservation was explored for over a decade. Ex situ protection is beneficial for expanding the population of animals and maintaining their genetic diversity; however, it can also induce metabolic diseases and parasitic infections and reduce reproductive capacity. The gut microbiota of animals has a considerable impact on host energy metabolism and immune regulation, thereby playing a crucial role in the overall health and reproductive success of the host. In this study, by comparing the differences in the intestinal microbiome of the musk deer according to their place of origin and migration, the changes in their gut microbiota and the influencing factors were explored to provide a theoretical basis for monitoring the health status of the musk deer. We used 16S rRNA high-throughput sequencing technology to analyze the structure and diversity of the gut microbiota of Alpine musk deer in Gansu (G, place of origin) and Sichuan (S, place of migration). The results showed that the dominant bacteria and genera in the intestinal microbiome of captive musk deer were similar in the places of origin and migration, but significant differences were observed in their relative abundance (*p* < 0.05). Regarding Firmicutes and Actinobacteria, which are related to plant cellulose digestion, the relative abundance in group G was higher than that in group S; regarding Proteobacteria and Verrucomicrobia, which are related to fat and starch intake, the relative abundance in group S was higher than that in group G; the relative abundance of *Bacillus* and *Clostridium sensu stricto*, which are related to fiber digestibility, was higher in group G than in group S; the relative abundance of conditional pathogens *Acinetobacter* and *Escherichia–Shigella* was higher in group S than in group G. The results of α and β diversity analysis also showed significant differences between the two groups (*p* < 0.05). The ACE and Shannon indices of musk deer in group G were considerably higher than those in group S, and the Simpson index of musk deer in group S was greater than that in group G, indicating that the abundance and diversity of intestinal microbiome were higher in musk deer of Gansu than those of Sichuan. Comparison of the changes in the intestinal microbiome of the musk deer according to the place of origin and migration showed that the plant cellulose content in the food of the musk deer, the fat content in the concentrated feed, and changes in the feeding environment have an impact on the intestinal microbiome. Effective monitoring of the health and immunity of the musk deer is crucial for ensuring their overall health, which in turn will aid in formulating a scientific and reasonable management plan for their conservation.

## 1. Introduction

The Alpine musk deer (*Moschus chrysogaster*), belonging to the family Moschidae, is endemic to China. It inhabits the alpine forest and shrub belt above 3000 m on the Qinghai–Tibet Plateau and the relatively cold and arid mountain forests extending to the northeast of the plateau [1]. Since the 1950s, China’s musk deer resources have drastically decreased by more than 90% [2]. However, the Alpine musk deer belongs to a relatively well-preserved species of Moschidae. In addition to the plateau’s forest shrub belt, Xinglong Mountain in Gansu Province (1159 individuals, [3,4]) and Helan Mountain in the Ningxia Hui Autonomous Region (100 individuals, [5,6]), which are located in the outer, mountainous regions of the plateau, maintain a certain population of musk deer. In the early 1990s, musk deer domestication and breeding began in the Xinglong Mountain area, which became the only source of the Alpine musk deer breeding population [7].

In 2006, the Chinese government promulgated a species breeding plan to establish breeding populations and actively promote reintroduction. Several years of the ex situ breeding practice have shown that the disease and death rates of the relocated population of the Alpine musk deer are higher than those of the original population, among which digestive tract diseases are particularly prominent. Studies have shown that animal digestive tract microbiota plays a vital role in host physiology, immunity, and metabolism [8,9,10].

An imbalance in intestinal microbiota is often accompanied by a reduction in microbiota diversity. The decreased diversity and richness of animal gut microbiota can increase the probability of diseases in the affected animals [11,12]. While gut microbiota is influenced by a variety of factors, food is the most direct factor affecting the diversity of animal intestinal microbiota [13]. Food is a substrate for microbial fermentation and drives the composition and metabolism of microbial communities [14].

Therefore, in this study, we compared the characteristics of the intestinal microbiota of the Alpine musk deer with the same provenance but different food sources and revealed the changes in the intestinal microbiota of ex situ Alpine musk deer and their possible relationship with digestive tract diseases. Our findings provide a scientific basis for improving the gut health of the Alpine musk deer and aid in devising management strategies for their conservation.

## 2. Materials and Methods

### 2.1. Experimental Environment and Experimental Animals

The Gansu musk deer (G musk deer) breeding base is located in Yuzhong County, which belongs to Xinglong Mountain in the eastward extension of the Qilian Mountains. It is 2171 m above sea level and has a temperate continental climate with an average annual temperature of 5.4 °C and an average annual precipitation of 406 mm. The Sichuan musk deer (S musk deer) breeding base is located in Qingchuan County and belongs to the northwestern section of Longmen Mountain in the Minshan Mountain System. It has an altitude of 1100 m and has a subtropical monsoon climate with an average annual temperature of 13.7 °C and an average annual precipitation of 1027 mm.

Eight healthy adult Alpine musk deer (8 male; 7–8 years old) were selected from both locations in mid-October 2018. Standardized construction of breeding centers, with all individuals raised separately. The breeding methods in the two locations were similar, with a captive breeding group structure. The main feeding plants of G musk deer included *Salix cupularis* Rehder, *Corylus mandshurica* Maxim. and Rupr., *Prunus salicina* Lindl, *Acer tetramerum* Pax, and *Prunus tomentosa* Thunb, whereas the main feeding plants of S musk deer included *Morus alba* L., *Ulmus pumila* L. cv. Tenue, *Styphnolobium japonicum* (L.) Schott and *Acer pictum* subsp. *mono* (Maxim.) H. Ohashi, *Broussonetia papyrifera* (Linn.) L’Hér. ex Vent., and *Eucommia ulmoides* Oliver. The food concentrations and ratios between the two places were the same, including corn starch, soybean, and bran.

### 2.2. Sampling and Experimentation

#### 2.2.1. Fresh Feces Collection

Each animal was sampled once, for a total of 16 fresh fecal samples. Disposable sterile gloves were worn during the sampling process, and fresh fecal samples were immediately placed in sterile centrifuge tubes for sealed storage. All samples were stored in liquid nitrogen and shipped back to the laboratory at the earliest time possible, where they were stored at −80 °C until DNA extraction.

#### 2.2.2. DNA Extraction, Polymerase Chain Reaction (PCR) Amplification, and 16S-rRNA Gene Sequencing

Bacterial DNA was extracted using MN NucleoSpin 96 Soi (Macherey-Nagel, Düren, Germany). DNA concentration and purity were measured using the Qubit dsDNA HS Assay Kit (Life Technologies, Carlsbad, CA, USA). The bacterial 16S rRNA gene fragments (V3–V4 region) were amplified from 16 fecal samples using the universal bacterial primers 338F (5′-ACTCCTACGGGAGGCAGCA-3′) and 806R (5′-GGACTACHVGGGTWTCTAAT-3′) [15]. The PCR volume was 10 μL, containing 5 μL KOD FX Neo Buffer, 0.2 μL KOD FX Neo, 2 μL dNTP, 10 μM forward and reverse primers at 0.3 μL each, with 50 ng ± 20% genomic DNA, and the remaining volume was comprised of ddH_2_O. The PCR conditions were as follows: 95 °C for 5 min, followed by 25 cycles of 95 °C for 30 s, 50 °C for 30 s, 72 °C for 40 s, and 72 °C for 7 min. According to the instructions for using the OMEGA DNA purification column, the PCR product was added to the purification column and collected and purified through repeated washing, elution, and centrifugation (Omega Bio tek, Nocross, GA, USA). Finally, high-throughput sequencing was performed on the Illumina HiSeq 2500 platform (Illumina, Inc., San Diego, CA, USA) at the Biomarker Technologies Corporation (Beijing, China).

#### 2.2.3. Bioinformatics and Statistical Analyses

The raw data were spliced (FLASH [16], version 1.2.11), the spliced sequences were subjected to quality filtering (Trimmomatic [17], version 0.33), and chimeras were removed (UCHIME [18], version 8.1) to obtain high-quality tag sequences. Sequences were clustered at the 97% similarity level, and 0.005% of all sequences was used as a threshold to filter out. The bacterial database was obtained from SILVA [19].

Mothur (version v.1.30.2) software was used to evaluate the Alpha diversity index of the samples and to analyze the richness and diversity index of bacterial communities in samples [20]. Microbial taxonomic compositions were shown as the mean ± SE. The statistical comparisons were made with the Student’s *t*-test. The level of significance (*p*) was set at 0.05. Analysis of variance (ANOVA) was used to test whether the difference of the α-diversity index between the two groups was significant. Alpha-diversity refers to the diversity within a specific region or ecosystem. Commonly used indicators to measure the abundance of microbiota include Chao1 and ACE richness estimators. Indicators used to measure diversity include the Shannon-Wiener diversity index and the Simpson diversity index. The smaller the value of the Simpson index, the higher the diversity of the community.

Beta diversity used QIIME 1.9.1 software to evaluate and analyze (https://qiime2.org/ (accessed on 11 December 2022)) [21]. Based on Bray-Curtis, a one-way analysis of similarities (ANOSIM) was performed to determine the differences between the groups. The vegan package in R version 4.2.2 (https://www.r-project.org (accessed on 11 December 2022)) was used for the analysis [22], and Python was used to construct the ANOSIM analysis graph. Linear discriminant analysis effect size (LEfSe) analysis, that is, the analysis of species with considerable differences between groups, uses linear discriminant analysis (LDA) to estimate the impact of the abundance of each species on the difference. It is mainly used to identify species with considerable differences in abundance between groups. A *t*-test was performed on the species abundance data between the groups to obtain the *p*-value, and finally the species that caused the difference in the composition of the two groups of the samples was screened out according to the *p*-value and the default *p* ≤ 0.05.

## 3. Results

### 3.1. 16srRNA Gene Sequencing Results

**Figure 1:**
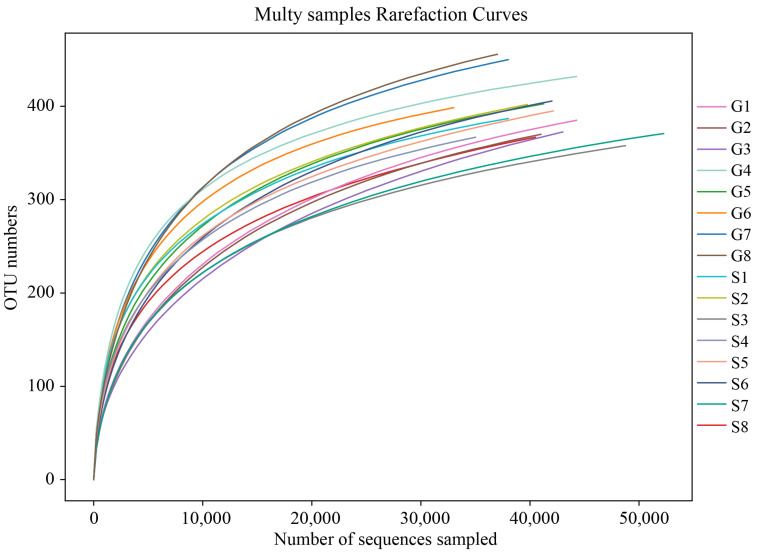
Rarefaction curve. The x-coordinate is the number of sequences sampled, and the y-coordinate is the number of observed OTUs. Each curve in the graph represents a sample, which is labeled with a different color. The number of OTUs increases with the sequencing depth. When the curve becomes stable, the number of detected OTUs does not increase with the expansion of extracted data, indicating a time when the amount of sequencing data is reasonable.

Based on Illumina MiSeq sequencing technology, this study amplified the 16S rRNA sequences of fecal microbiome and obtained 1,279,555 sequences (raw reads) from 16 samples of Alpine musk deer from Gansu and Sichuan, including 909,531 effective sequences (effective reads). An average of 56,845.69 effective sequences were obtained per sample (the average sequence length (AvgLen) was 423.94 bp). A total of 608 operational taxonomic units (OTUs) were obtained at the 97% sequence similarity level, with an average of 395.06 OTUs per sample. As the sequencing depth increased, the number of OTUs increased, and the OTU dilution curve measured in this study (Figure 1) eventually flattened, indicating that the amount of sequencing data was reasonable.

**Figure 2:**
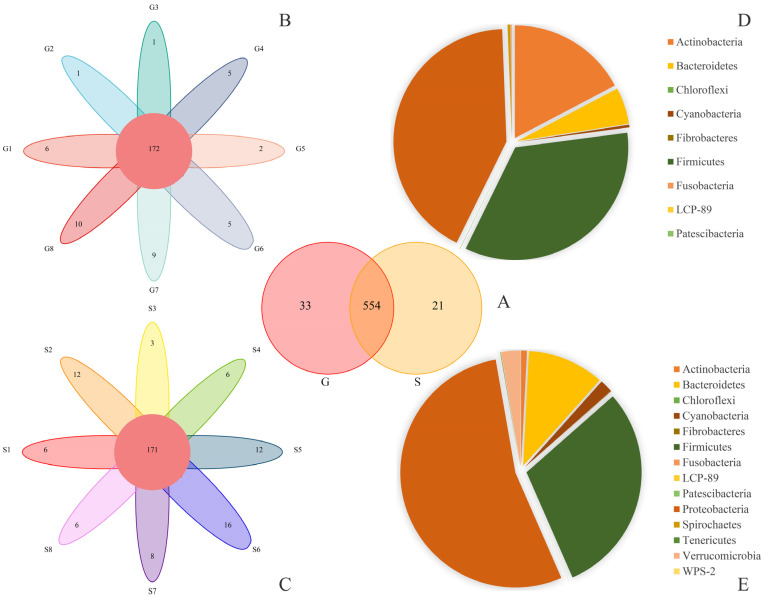
OTU Venn diagram, flower diagram, and microflora structure (phylum level) pie chart. The Venn and flower diagrams show the overlapping number of multiple color graphs, which refers to the total number of OTUs shared by G and S individuals, and the non-overlapping number, which refers to the unique number of OTUs in each sample. The pie diagrams show different colored blocks for different types of microbiotas (phylum level), and the numbers on the colored blocks represent the percentage of the relative abundance of microbiota. (**A**) The number of OTUs shared by G and S. (**B**) The number of OTUs shared within G. (**C**) The number of OTUs shared within S. (**D**) The proportion of phylum relative abundance of S. (**E**) The proportion of phylum relative abundance of G.

The Venn and petal diagrams show shared and unique bacterial species in the fecal samples of Alpine musk deer from Gansu and Sichuan, and the bacterial taxa shared by all animals in both groups were regarded as the core microbiota in the hindgut of Alpine musk deer. There were 554 OTUs in the fecal samples of Gansu and Sichuan musk deer, with 33 OTUs in the former and 21 OTUs in the latter (Figure 2A); there were 172 OTUs in Gansu musk deer and 171 OTUs in Sichuan musk deer (Figure 2B,C). Using a microbial reference database, the main bacteria detected were classified into 14 phyla, 22 classes, 40 orders, 83 families, and 191 genera.

**Figure 3:**
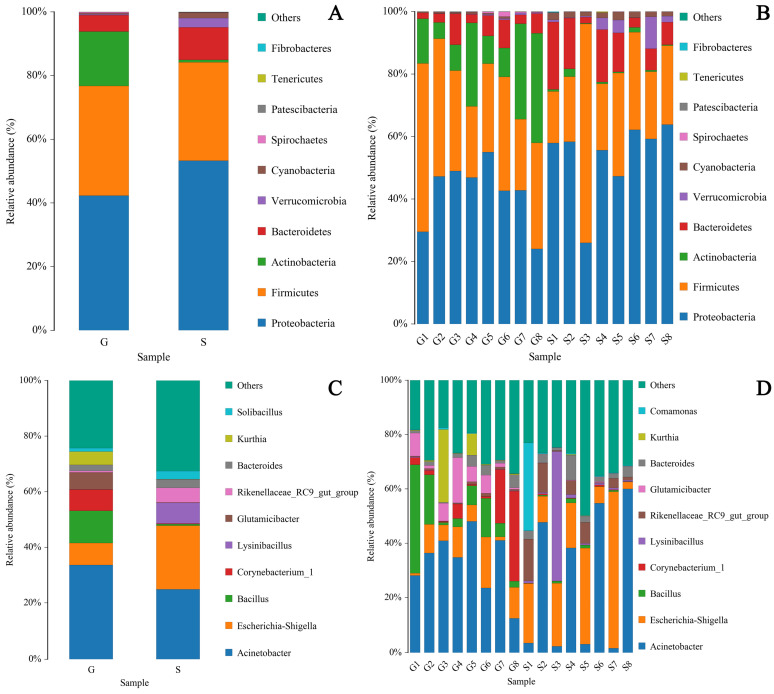
Core microbiota (phylum and genus level) distribution histogram. The x-coordinate represents sample and group name, and the y-coordinate represents relative abundance percentage. A color represents a species, and color block length (bar chart) indicates the relative abundance ratio of the species. The “Others” category represents other species in the top ten with respect to the abundance level, and “Unclassified” represents species that have not been classified. (**A**,**B**) represent core flora distribution at the phylum level between G and S groups. (**C**,**D**) represent core flora distribution at the genus level between G and S groups.

The pie chart shows the proportions of 14 major bacterial phyla in Gansu and Sichuan Alpine musk deer fecal samples (Figure 2D,E), while the distribution histogram visually shows the relative abundance of the top ten bacterial phyla (Figure 3A,C) and the proportion of bacterial genera (Figure 3B,D) in the fecal microbiome of the two regions. The top ten phyla in terms of relative abundance were *Proteobacteria* (G 42.12%; S 53.79%), *Firmicutes* (G 34.31%; S 29.97%), *Actinobacteria* (G 17.28%; S 0.82%), *Bacteroidetes* (G 5.24%; S 10.78%), *Cyanobacteria* (G 0.37%; S 1.85%), *Spirochaetes* (G 0.34%; S 0.04%), *Verrucomicrobia* (G 0.24%; S 2.66%), *Acidobacteria Patescibacteria* (G 0.05%; S 0.01%), *Tenericutes* (G 0.02%; S 0.04%), and *Fibrobacteres* (G 0.01%; S 0.03%).

The top ten genera in terms of relative abundance were *Acinetobacter* (G 33.16%; S 26.39%), *Bacillus* (G 11.34%; S 0.59%), *Escherichia-Shigella* (G 8.23%; S 21.50%), *Corynebacterium_1* (G 8.00%; S 0.22%), *Glutamicibacter* (G 5.88%; S 0.09%), *Kurthia* (G 4.54%; S 0.05%), *uncultured_bacterium_f_Planococcaceae* (G 3.12%; S 0.76%), *Clostridium_sensu_stricto_1* (G 1.03%; S %), *Arthrobacter* (G 2.39%; S 0.13%), and *Planomicrobium* (G 2.32%; S 0.01%).

### 3.2. Analysis of Intestinal Microbiome Diversity

**Figure 4:**
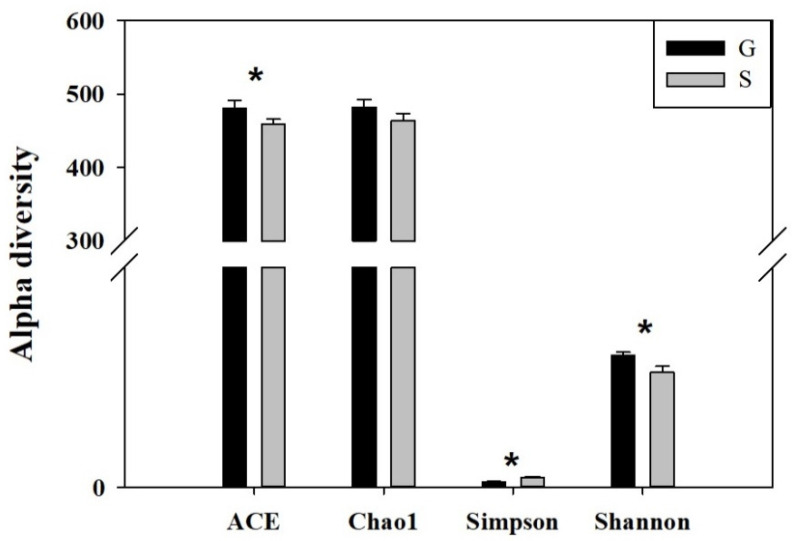
Inter-group analysis of variance (ANOVA) histogram of α-diversity index. ACE: an index used to estimate the number of OTUs in a community; Chao 1: an index that uses the Chao1 algorithm to estimate the number of OTUs included in a sample. ACE and Chao 1 are commonly used in ecology to assess the total number of species; Shannon: an index used to estimate microbial diversity in a sample; Simpson: an index used to quantitatively describe the biodiversity of a geographical area. * *p* < 0.05.

In this study, different indicators were used to compare the diversity of fecal samples from the Alpine musk deer in Gansu and Sichuan. α-diversity reflects species richness and species diversity of a single sample. Chao1 and ACE indices were used to measure species abundance. There was no significant difference in the Chao1 index of the intestinal microbiome between the Gansu and Sichuan Alpine musk deer, but there was a significant difference in their ACE index (*p* < 0.05) (Figure 4). Shannon and Simpson indices were used to measure species diversity; there were significant differences in the Shannon and Simpson indices of the intestinal microbiome of musk deer between the two regions (*p* < 0.05) (Figure 4). For the same species abundance, the greater the community evenness and the greater the species diversity of the community were; that is, the greater the Shannon index value, the smaller the Simpson index value and the higher the species diversity of the sample were (see Table 1 below). These results showed that the species diversity of the intestinal microbiome of musk deer in Gansu was higher than that in Sichuan. The integrity of the sequencing was tested using Good’s coverage, which was close to 99% in this study, indicating that most of the bacterial species present in the samples had been detected.

**Figure 5:**
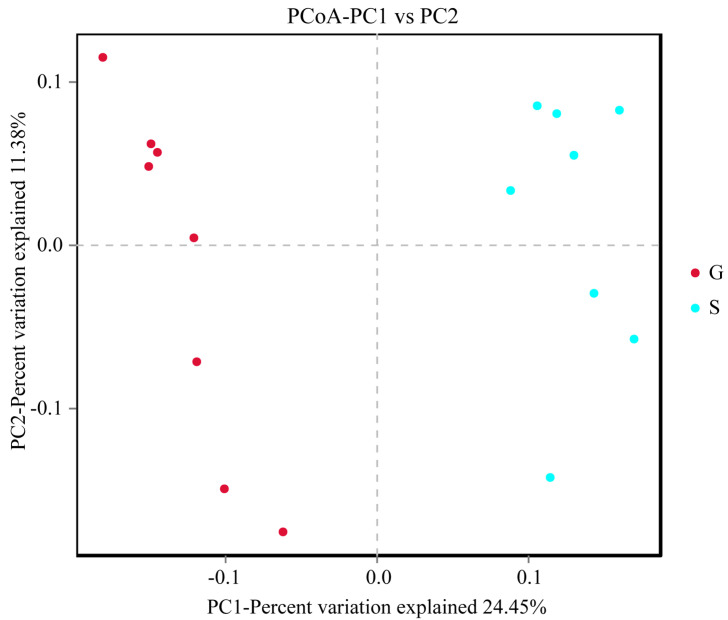
Principal coordinate analysis (PCoA) plot of β-diversity. Samples in the same group are represented by the same color and shape. PC1 vs. PC2 is the PCoA plot obtained from the first and second main coordinates; the x-axis and y-axis represent the first and second main coordinates, respectively. The percentage of the main coordinates represents the relative contribution of this coordinate to sample differences, which is a measure of the amount of original information extracted by this main coordinate. The distances between the sample points represent the similarity of microbiota in the samples. A closer distance represents higher similarity; samples that cluster together are composed of similar microbiota.

**Figure 6:**
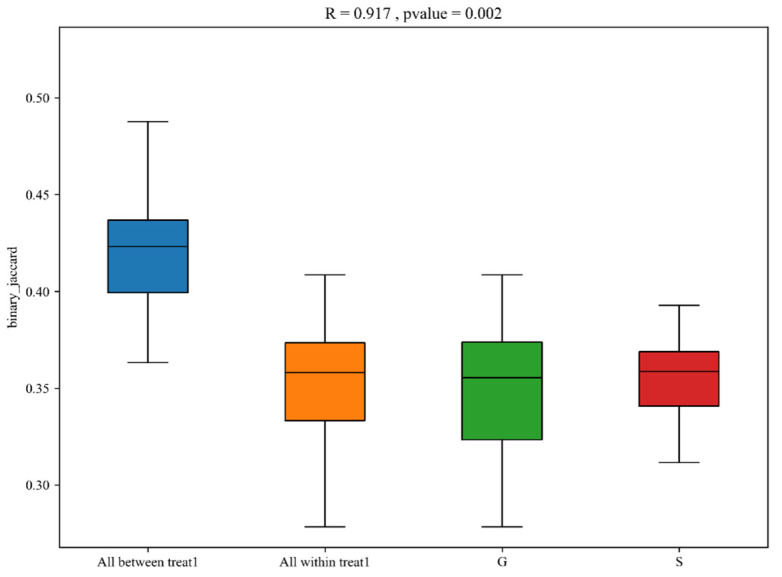
Inter-group analysis of similarities (ANOSIM) boxplot of β-diversity. The *y*-axis represents the rank of the distance between samples, and the *x*-axis represents the results between both groups. Intra-group results are shown for each group. R value: R-value range (−1, 1). Actual results are generally between 0 and 1. An R-value close to 0 represents no significant inter-group and intra-group differences. An R-value close to 1 shows that inter-group differences are greater than intra-group differences. *p*-value: The *p*-value represents the confidence level of the statistical analysis; *p* < 0.05 reflects a statistically significant difference. In the plot, the R-value was close to 1, indicating that inter-group differences were greater than the intra-group differences, and *p* < 0.05 shows that this result was statistically significant.

QIIME 1.9.1 software was used to perform β-diversity analysis, reflecting the differences in species diversity among populations by comparing temporal and spatial changes in microbial composition. Principal coordinate analysis (PCoA) is a dimensionality reduction sorting method that uses the difference or distance between the samples to represent differences in species diversity, achieving a quantitative conversion of qualitative data. The closer the distance on the PC1 vs. PC2 coordinate map, the greater the similarity of the samples. The results of PCoA analysis of β-diversity showed that the intestinal microbiome of Gansu and Sichuan Alpine musk deer can be clearly clustered into two categories (Figure 5). ANOSIM was used to determine significant differences in β-diversity between samples from different groups. In this study, the vegan package in R was used for ANOSIM analysis. An R-value close to 1 indicates that the difference between the groups is greater than that within a group, whereas a smaller R-value indicates that there is no considerable difference between groups or within a group. The results of ANOSIM analysis of β-diversity showed that the differences between groups in the intestinal microbiome of the Alpine musk deer in the two regions were significantly higher than the differences within the groups (R = 0.917, *p* < 0.05) (Figure 6).

### 3.3. Differences in Microbiome Composition

**Figure 7:**
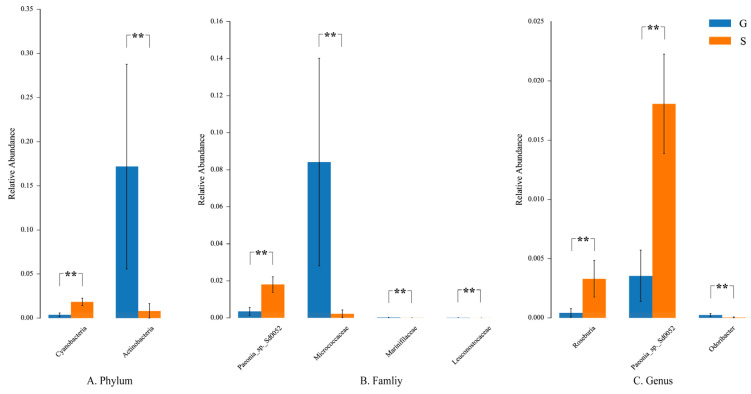
ANOVA of inter-group samples. The plot from ANOVA shows differences in relative abundance (mean% ± SD) of two bacterial phyla, four bacterial families, and three bacterial genera. ** *p* < 0.01. (**A**) Relative abundance at the phylum level. (**B**) Relative abundance at the family level. (**C**) Relative abundance at the genus level.

Analysis of variance (ANOVA) was used to test the significance of difference in means among multiple samples. At the phylum level, there were significant differences in Actinobacteria and Cyanobacteria between the fecal microbiota of Gansu and Sichuan musk deer. The relative abundance of Cyanobacteria in group S was greater than that in group G, and the relative abundance of Actinobacteria in group G was greater than that in group S. At the family level, the relative abundance of *Micrococcaceae*, *Marinifilaceae*, and *Leuconostocaceae* in group G was higher than those in group S. At the genus level, the relative abundance of *Odoribacter* in group G was higher than that in group S, and the relative abundance of *Paeonia* sp. Sd0052 and *Roseburia* was higher in group S than in group G (*p* < 0.05) (Figure 7).

**Figure 8:**
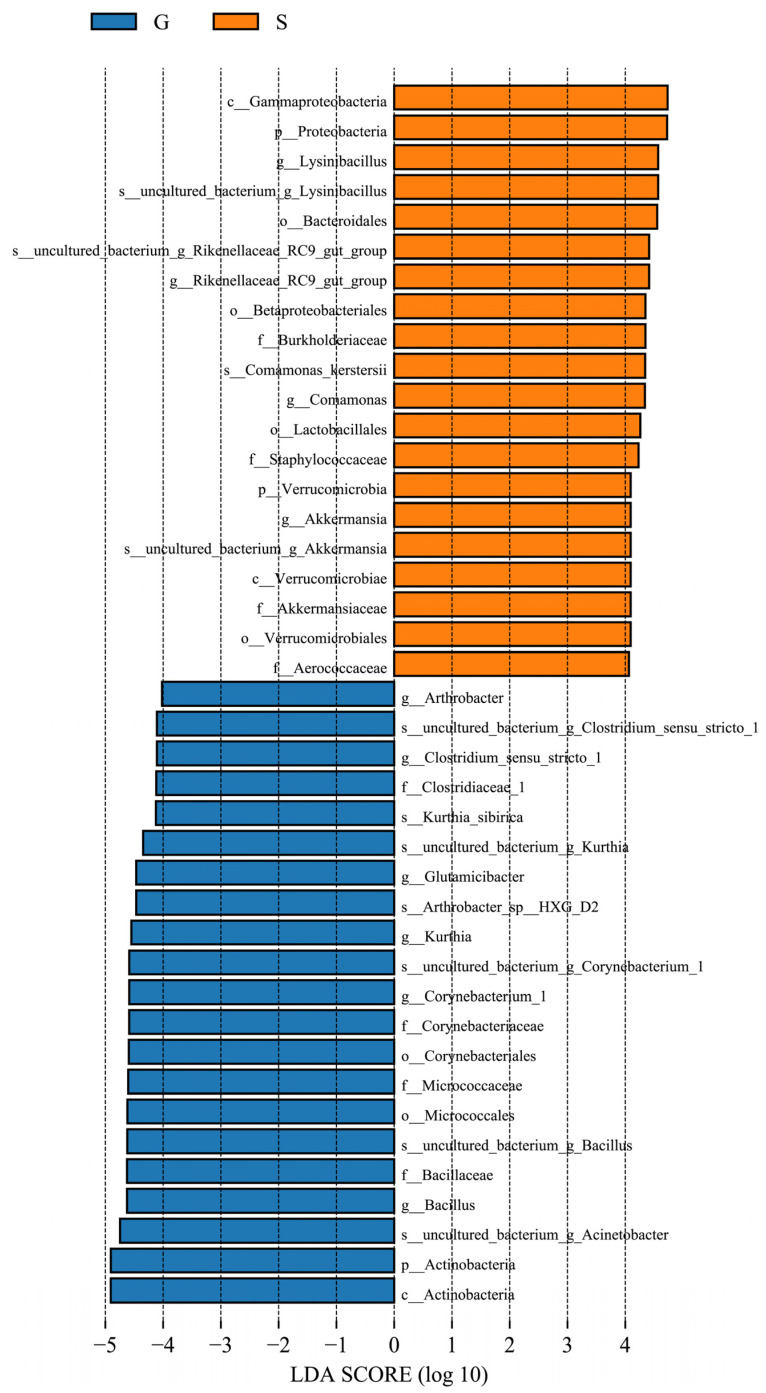
Linear discriminant analysis (LDA) effect size (LEfSe) of inter-group samples. The length of the bar column represents the LDA score, and different colors represent different groups of species. The plot from LEfSe analysis shows the microbial taxa with significant differences between G (blue) and S (orange) (LDA score > 4).

LEfSe can identify biomarkers with statistical differences among different groups. The length of the histogram represents the LDA score, which, in turn, represents the impact of different species. LEfSe analysis identified 41 bacterial taxa that were significantly different between the two groups of gut microbiota of the musk deer. The relative abundance of *Actinobacteria*, *Bacillus*, *Corynebacterium 1*, *Kurthia*, *Glutamicibacter*, *Clostridium sensu stricto 1*, and *Arthrobacter* was considerably higher in group G than in group S. The relative abundance of Proteobacteria, Verrucomicrobia, *Lysinibacillus*, *RikenellACEae RC9* gut group, *Comamonas*, and *Akkermansia* was considerably higher in group S than in group G (Figure 8).

## 4. Discussion

Ex situ conservation is an important technique for protecting rare and endangered wildlife that enables population recovery and rapid expansion. To reduce the risk of maladaptive factors in animals due to ex situ conservation, their health status should be continuously monitored. Intestinal health is a vital factor regulating health, and the dynamic equilibrium between intestinal microorganisms and the body affects digestion, absorption of nutrients, metabolism, and immunity [23,24]. Imbalances in intestinal microbiota can cause several serious diseases and severe damage to the body [25,26].

Studies have shown that microbial diversity can reflect the health status of animals to a certain extent [27], and that low intestinal microbiome diversity and a simple mi-crobial community structure may cause a variety of diseases [27]. In contrast, high micro-bial diversity indicates stronger intestinal stability [28]. Instability of the microbial com-munity is a health hazard for the host [29]. In this study, there were significant differences in the α- and β-diversities of the intestinal microbiome of Alpine musk deer in Gansu and Sichuan. The abundance and diversity of the intestinal microbiome of Gansu Alpine musk deer were higher than those of Sichuan Alpine musk deer. The health status of Gansu Alpine musk deer was better than that of Sichuan Alpine musk deer.

Firmicutes and Actinobacteria in the intestinal microbiome of herbivores are associated with the digestion of plant cellulose [30,31]. The abundance of Firmicutes and Actinobacteria in the musk deer in group G was higher than those in the musk deer in group S (Figure 3), reflecting the higher cellulose content in the food of Alpine musk deer in Gansu owing to the presence of rougher plants in the musk deer’s native area. A previous study found that the relative abundance of Firmicutes in wild Alpine musk deer feces was considerably higher than that in captive musk deer, whereas that of Bacteroidetes was considerably lower than that in captive musk deer [32]. *Bacillus* and *Clostridium sensu stricto* in Firmicutes can improve the digestibility of high-fiber diets [33,34]. The abundance of *Bacillus* and *Clostridium sensu stricto* in group G was higher than that in group S (Figure 8). The cellulose of food source plants is the main factor responsible for the differences in the abundance of Firmicutes and Actinobacteria. Proteobacteria and Verrucomicrobia were negatively correlated with fiber intake [35,36] but positively correlated with fat and starch intake [36,37]. The abundance of Proteobacteria and Verrucomicrobia in group S was higher than that in group G (Figure 3), which reflected that the Sichuan Alpine musk deer consumed plants with more nutrients such as carbohydrates and lipids.

Additionally, the proliferation of Proteobacteria can cause metabolic disorders [38,39], and high-fat diets can lead to a considerable increase in abundance of Proteobacteria in the intestinal tract of mice [40]. The abundance of Proteobacteria in the intestinal tract of the Sichuan Alpine musk deer was higher (Figure 3), which shows that compared with the place of origin Gansu, the subtropical plants ingested by the Alpine musk deer in group S had higher fat content. Both *Acinetobacter* and *Escherichia*–*Shigella* under the phylum Proteobacteria are opportunistic pathogenic bacteria [41,42], which had higher abundance in the musk deer in group S (Figure 3) and can cause intestinal diseases through food. The high relative abundance of Proteobacteria is an important reason for imbalance in the intestinal microbial flora of animals [39] and can lead to the increase of pathogenic bacteria, thereby adversely affecting the health of the host [27,43]. Proteobacteria are the dominant bacteria in the intestines of white-tailed deer [44] and American bison [45]. However, the relative abundance of Proteobacteria in the two groups in the present study was higher than that in other ruminants [46,47,48], which may be related to the artificial addition of high-fat concentrate feed to musk deer farms.

Actinobacteria are vital not only for maintaining the homeostasis of the intestinal barrier [49] but also for regulating the host health and enhancing the immune function [50]. Studies have found that Actinobacteria can regulate the bile acid content and virulence of intestinal pathogens, which is beneficial to the intestinal health of the host [51]. Actinobacteria and their genera are mostly beneficial to the host because they produce active metabolites, such as antibiotics, enzymes, and antitumor substances in the gut [52], which can inhibit and eliminate most pathogens in the animal gut [53]. *Bacillus* improves flora imbalance and anti-intestinal inflammation and helps to reduce intestinal mucosal damage caused by diseases. Some strains are also used to treat metabolic disorders [54]. Supplementation of animal feed with specific strains of *Bacillus* promotes digestion, growth performance, microbiota modulation, and immune function [55]. The abundance of Actinobacteria and *Bacillus* in group G was higher than that in group S (Figure 3), which indicated that the musk deer was more adapted to living conditions at the place of origin, as they were conducive to maintaining its intestinal homeostasis and immunity.

Ex situ conservation is an important means of protecting endangered species [56], but it also leads to a series of issues [21]. Artificial feeding may cause wild animals to lose their original gut microbes [22,57], thus reducing the diversity and cellulose degradation capacity of the microbes [58]. Long-term captivity affects the adaptability of animals in the wild. In order to rapidly restore the population size of the Alpine musk deer and achieve wild reintroduction, scientifically formulating a comprehensive conservation plan and promoting its ex situ conservation should become the main trend in the future. Our research data reveal the importance of dietary differences in the migration Alpine musk deer sites. By optimizing the diet of the Alpine musk deer, we can increase the richness of its intestinal microbial community, thereby ensuring intestinal health during the process of breeding in different places. The implementation of this strategy will help researchers to explore ways to improve survival rates, thus providing necessary technical support for population recovery.

## 5. Conclusions

In summary, it is of great significance to maintain the diversity and stability of the gut microbiota of captive Alpine musk deer after migration. This study utilizes gut microbiota to monitor the health status of the population, providing a theoretical basis for expanding artificial populations of Alpine musk deer and reintroducing wild populations. However, in order to comprehensively understand the health status of the migrant population, further research is needed to combine factors such as feeding status and activity level of Alpine musk deer. By comprehensively analyzing these factors, we can more accurately assess the health status of Alpine musk deer populations and take corresponding conservation measures to promote their population recovery and wild reintroduction.

## Figures and Tables

**Table 1:** Statistics of α-diversity indices.

Group	Chao1	ACE	Shannon	Simpson
G	482.33 ± 10.58	481.16 ± 10.17	2.99 ± 0.08	0.13 ± 0.01
S	463.53 ± 10.39	458.69 ± 6.98	2.61 ± 0.14	0.22 ± 0.03
P	>0.05	<0.05	<0.05	<0.05

## Data Availability

The sequencing data generated in our study have been deposited in the SRA database and the BioProject ID is PRJNA1036391.

## References

[1] Wu J., Wang W. (2006). The Musk Deer of China.

[2] Sheng H.L., Liu Z.X. (2007). The Musk Deer in China.

[3] Wang J., Sun J.P., Xu T., Qi J., Zhang Y.L., Zhang X.Y., Meng X.X. (2009). Population distribution, quantitative characteristics and influencing factors of the wild alpine musk deer in Xinglongshan National Nature Reserve, Gansu Province. Acta Ecol. Sin..

[4] Gao H.X., Shen L.Q., Liu R., Wang G., Zhang A.P., Chen L., Zhang Y.Z., Zhang X.Y., Qi J., Wang C.L. (2023). Population Dynamics of Alpine Musk Deer in Xinglongshan National Nature Reserve and the Relationships to the Summer Habitat Suitability. Chin. J. Wildl..

[5] Yang Y.T., Yang Y.Y., Na H.Y., Deng E., Yuan L.L. (2017). Study on the survival status of alpine musk deer on the western slope of Helan Mountain. Agr. Tech..

[6] Ding Y.K., Yao Z.C., Zhao C., Zhang Z.R., Chen J.D., Teng L.W., Liu Z.S. (2023). Habitat suitability assessment of *Moschus chrysogaster* in Helan Moutain. Acta Ecol. Sin..

[7] Liu Z.X., Li Q., Kang F.G., Sheng H.L. (2001). Some ecological characteristics of the isolated population of alpine musk deer (*Moschus chrysogaster*) in the Xinglong forest, Gansu Province. Acta Ecol. Sin..

[8] Hooper L.V., Littman D.R., Macpherson A.J. (2012). Interactions between the microbiota and the immune system. Science.

[9] Warne R.W., Kirschman L., Zeglin L. (2019). Manipulation of gut microbiota during critical developmental windows affects host physiological performance and disease susceptibility across ontogeny. J. Anim. Ecol..

[10] Zhu L., Wu Q., Dai J., Zhang S., Wie F. (2011). Evidence of cellulose metabolism by the giant panda gut microbiome. Proc. Natl. Acad. Sci. USA.

[11] Li Y.M., Hu X.L., Yang S., Zhou J.T., Qi L., Sun X.N., Fan M.Y., Xu S.H., Cha M.H., Zhang M.S. (2018). Comparison Between the Fecal Bacterial Microbiota of Healthy and Diarrheic Captive Musk Deer. Front. Microbiol..

[12] Zhao W., Ren Z.W., Luo Y., Cheng J.G., Wang J., Wang Y., Yang Z.X., Yao X.P., Zhong Z.J., Yang W. (2021). Metagenomics analysis of the gut microbiome in healthy and bacterial pneumonia forest musk deer. Genes Genom..

[13] Hicks A.L., Lee K.J., Couto-Rodriguez M., Patel J., Sinha R., Guo C., Olson S.H., Seimon A., Seimon T.A., Ondzie A.U. (2018). Gut microbiomes of wild great apes fluctuate seasonally in response to diet. Nat. Commun..

[14] Muegge B.D., Kuczynski J., Knights D., Clemente J.C., Gonzalez A., Fontana L., Henrissat B., Knight R., Gordon J.I. (2011). Diet Drives Convergence in Gut Microbiome Functions Across Mammalian Phylogeny and Within Humans. Science.

[15] Dennis K.L., Wang Y., Blatner N.R., Wang S., Saadalla A., Trudeau E., Roers A., Weaver C.T., Lee J.J., Gilbert J.A. (2013). Adenomatous polyps are driven by microbe-instigated focal inflammation and are controlled by il-10–producing T cells. Cancer Res..

[16] Magoc T., Salzberg S.L. (2011). FLASH: Fast length adjustment of short reads to improve genome assemblies. Bioinformatics.

[17] Bolger A.M., Lohse M., Usadel B. (2014). Trimmomatic: A flexible trimmer for Illumina sequence data. Bioinformatics.

[18] Edgar R.C., Haas B.J., Clemente J.C., Quince C., Knight R. (2011). UCHIME improves sensitivity and speed of chimera detection. Bioinformatics.

[19] Quast C., Pruesse E., Yilmaz P., Gerken J., Schweer T., Yarza P., Peplies J., Glockner F.O. (2013). The SILVA ribosomal RNA gene database project: Improved data processing and webbased tools. Nucleic Acids Res..

[20] Schloss P.D., Westcott S.L., Ryabin T., Hall J.R., Hartmann M., Hollister E.B., Lesniewski R.A., Oakley B.B., Parks D.H., Robinson C.J. (2009). Introducing Mothur: Open-source, platform-independent, community-supported software for describing and comparing microbial communities. AEM.

[21] Williams C.L., Caraballo-Rodríguez A.M., Allaband C., Zarrinpar A., Knight R., Gauglitz J.M. (2018). Wild-life-microbiome interactions and disease: Exploring opportunities for disease mitigation across ecological scales. Drug Discov. Today.

[22] Gibson K.M., Nguyen B.N., Neumann L.M., Miller M., Buss P., Daniels S., Ahn M.J., Crandall K.A., Pukazhenthi B. (2019). Gut microbiome differences between wild and captive black rhinoceros–implications for rhino health. Sci. Rep..

[23] Nicholson J.K., Holmes E., Kinross J., Burcelin R., Gibson G., Jia W., Pettersson S. (2012). Host-gut microbiota metabolic interactions. Science.

[24] Ottman N., Smidt H., De Vos W.M., Belzer C. (2012). The function of our microbiota: Who is out there and what do they do?. Front. Cell Infect Microbiol..

[25] Houttu V., Boulund U., Grefhorst A., Soeters M.R., Pinto-Sietsma S., Nieuwdorp M., Holleboom A.G. (2020). The role of the gut microbiome and exercise in non-alcoholic fatty liver disease. Therap. Adv. Gastroenterol..

[26] Wu J., Wang K., Wang X., Pang Y., Jiang C. (2021). The role of the gut microbiome and its metabolites in metabolic diseases. Protein Cell..

[27] Jayachandran M., Chung S.S.M., Xu B. (2020). A critical review of the relationship between dietary components, the gut microbe Akkermansia muciniphila, and human health. Crit. Rev. Food Sci. Nutr..

[28] Lozupone C.A., Stombaugh J.I., Gordon J.I., Jansson J.K., Knight R. (2012). Diversity, stability and resilience of the human gut microbiota. Nature.

[29] Mosca A., Leclerc M., Hugot J.P. (2016). Gut microbiota diversity and human diseases: Should we reintroduce key predators in our ecosystem?. Front. Microbiol..

[30] Jiang F., Gao H.M., Qin W., Song P.F., Wang H.J., Zhang J.J., Liu D.X., Wang D., Zhang T.Z. (2021). Marked Seasonal Variation in Structure and Function of Gut Microbiota in Forest and Alpine Musk Deer. Front. Microbiol..

[31] Berlemont R., Martiny A.C. (2013). Phylogenetic distribution of potential cellulases in bacteria. AEM.

[32] Sun Y.W., Sun Y.J., Shi Z.H., Liu Z.S., Zhao C., Lu T.F., Gao H., Zhu F., Chen R., Zhang J. (2020). Gut microbiota of wild and captive Alpine musk deer (*Moschus chrysogaster*). Front. Microbiol..

[33] Singh A., Tiwari U., Berrocoso J., Dersjant-Li Y., Awati A., Jha R. (2019). Effects of a combination of xylanase, amylase and protease, and probiotics on major nutrients including amino acids and non-starch polysaccharides utilization in broilers fed different level of fibers. Poult. Sci..

[34] Kong C., Gao R.Y., Yan X.B., Huang L.S., Qin H.L. (2019). Probiotics improve gut microbiota dysbiosis in obese mice fed a high-fat or high-sucrose diet. Nutrition.

[35] Zhang Z.X., Taylor L., Shommu N., Ghosh S., Reimer R., Panaccione R., Kaur S., Hyun J.E., Cai C., Deehan E.C. (2020). A diversified dietary pattern is associated with a balanced gut microbial composition of Faecalibacterium and Escherichia/Shigella in patients with Crohn’s disease in remission. J. Crohns Colitis..

[36] Zhu Y.L., Wang C.Y., Li F.C. (2015). Impact of dietary fiber/starch ratio in shaping caecal microbiota in rabbits. Can. J. Microbiol..

[37] Méndez-Salazar E.O., Ortiz-López M.G., Granados-Silvestre M.Á., Palacios-González B., Menjivar M. (2018). Altered gut microbiota and compositional changes in Firmicutes and Proteobacteria in Mexican undernourished and obese children. Front. Microbiol..

[38] Fei N., Zhao L.P. (2013). An opportunistic pathogen isolated from the gut of an obese human causes obesity in germfree mice. ISME J..

[39] Shin N.R., Whon T.W., Bae J.W. (2015). Proteobacteria: Microbial signature of dysbiosis in gut microbiota. Trends Biotechnol..

[40] Jeong M.Y., Jang H.M., Kim D.H. (2019). High-fat diet causes psychiatric disorders in mice by increasing Proteobacteria population. Neurosci. Lett..

[41] Carvalheira A., Silva J., Teixeira P. (2020). *Acinetobacter* spp. in food and drinking water—A review. Food Microbiol..

[42] Khba B., Jdaia C., Fm D. (2020). Heterogeneity in enterotoxigenic Escherichia coli and shigella infections in children under 5 years of age from 11 African countries: A subnational approach quantifying risk, mortality, morbidity, and stunting. Lancet Glob. Health.

[43] Althani A.A., Marei H.E., Hamdi W.S., Nasrallah G.K., El Zowalaty M.E., Al Khodor S., Al-Asmakh M., Abdel-Aziz H., Cenciarelli C. (2016). Human microbiome and its association with health and diseases. J. Cell Physiol..

[44] Delgado M.L., Singh P., Funk J.A., Moore J.A., Cannell E.M., Kanesfsky J., Manning S.D., Scribner K.T. (2017). Intestinal microbial community dynamics of white-tailed deer (*Odocoileus virginianus*) in an agroecosystem. Microb. Ecol..

[45] Weese J.S., Shury T., Jelinski M.D. (2014). The fecal microbiota of semi-free-ranging wood bison (*Bison bison athabascae*). BMC Vet. Res..

[46] Hu X.L., Liu G., Shafer A.B.A., Wei Y.T., Zhou J.T., Lin S.B., Wu H.B., Mi Z., Hu D.F., Liu S.Q. (2017). Comparative analysis of the gut microbial communities in forest and alpine musk deer using high-throughput sequencing. Front. Microbiol..

[47] Li Y.M., Hu X.L., Yang S., Zhou J.T., Zhang T.X., Qi L., Sun X.N., Fan M.Y., Xu S.H., Cha M.H. (2017). Comparative Analysis of the Gut Microbiota Composition between Captive and Wild Forest Musk Deer. Front. Microbiol..

[48] Sawaswong V., Praianantathavorn K., Chanchaem P., Khamwut A., Kemthong T., Hamada Y., Malaivijitnond S., Payungporn S. (2021). Comparative analysis of oral-gut microbiota between captive and wild long-tailed macaque in Thailand. Sci. Rep..

[49] Binda C., Lopetuso L.R., Rizzatti G., Gibiino G., Cennamo V., Gasbarrini A. (2018). Actinobacteria: A relevant minority for the maintenance of gut homeostasis. Dig. Liver Dis..

[50] Kim J.H., Kim Y., Kim Y.J., Park Y. (2016). Conjugated linoleic acid: Potential health benefits as a functional food ingredient. Annu. Rev. Food Sci. Technol..

[51] Jeong Y., Kim J.W., You H.J., Park S.J., Lee J., Ju J.H., Park M.S., Jin H., Cho M.L., Kwon B. (2019). Gut microbial composition and function are altered in patients with early rheumatoid arthritis. J. Clin. Med..

[52] Sun R.L., Xu K., Ji S.B., Pu Y.Q., Man Z.D., Ji J.H., Chen M.J., Yin L.H., Zhang J., Pu Y.P. (2020). Benzene exposure induces gut microbiota dysbiosis and metabolic disorder in mice. Sci. Total Environ..

[53] Weber T., Charusanti P., Musiol-Kroll E.M., Jiang X., Tong Y., Kim H.U., Lee S.Y. (2015). Metabolic engineering of antibiotic factories: New tools for antibiotic production in actinomycetes. Trends Biotechnol..

[54] Elshaghabee F.M., Rokana N., Gulhane R.D., Sharma C., Panwar H. (2017). Bacillus as potential probiotics: Status, concerns, and future perspectives. Front. Microbiol..

[55] Bernardeau M., Lehtinen M.J., Forssten S.D., Nurminen P. (2017). Importance of the gastrointestinal life cycle of Bacillus for probiotic functionality. J. Food Sci. Technol..

[56] West A.G., Waite D.W., Deines P., Bourne D.G., Digby A., McKenzie V.J., Taylor M.W. (2019). The microbiome in threatened species conservation. Biol. Conserv..

[57] Tsukayama P., Boolchandani M., Patel S., Pehrsson E.C., Gibson M.K., Chiou K.L., Jolly C.J., Rogers J., Phillips-Conroy J.E., Dantas G. (2018). Characterization of wild and captive baboon gut microbiota and their antibiotic resistomes. Msystems.

[58] Guo W., Mishra S., Wang C.D., Zhang H.M., Ning R.H., Kong F.L., Zeng B., Zhao J.C., Li Y. (2019). Comparative study of gut microbiota in wild and captive giant pandas (*Ailuropoda melanoleuca*). Genes.

